# An Age-Adjusted EEG Source Classifier Accurately Detects School-Aged Barbadian Children That Had Protein Energy Malnutrition in the First Year of Life

**DOI:** 10.3389/fnins.2019.01222

**Published:** 2019-11-29

**Authors:** Maria L. Bringas Vega, Yanbo Guo, Qin Tang, Fuleah A. Razzaq, Ana Calzada Reyes, Peng Ren, Deirel Paz Linares, Lidice Galan Garcia, Arielle G. Rabinowitz, Janina R. Galler, Jorge Bosch-Bayard, Pedro A. Valdes Sosa

**Affiliations:** ^1^The Clinical Hospital of Chengdu Brain Sciences, University of Electronic Science and Technology of China, Chengdu, China; ^2^Cuban Neuroscience Center, Havana, Cuba; ^3^Department of Neurology and Neurosurgery, McGill University, Montreal, QC, Canada; ^4^Division of Pediatric Gastroenterology and Nutrition, Massachusetts General Hospital for Children, Boston, MA, United States; ^5^Montreal Neurological Institute, McGill University, Montreal, QC, Canada

**Keywords:** protein energy malnutrition PEM, children, EEG, source analysis, classifiers

## Abstract

We have identified an electroencephalographic (EEG) based statistical classifier that correctly distinguishes children with histories of Protein Energy Malnutrition (PEM) in the first year of life from healthy controls with 0.82% accuracy (area under the ROC curve). Our previous study achieved similar accuracy but was based on scalp quantitative EEG features that precluded anatomical interpretation. We have now employed BC-VARETA, a novel high-resolution EEG source imaging method with minimal leakage and maximal sparseness, which allowed us to identify a classifier in the source space. The EEGs were recorded in 1978 in a sample of 108 children who were 5–11 years old and were participants in the 45+ year longitudinal Barbados Nutrition Study. The PEM cohort experienced moderate-severe PEM limited to the first year of life and were age, handedness and gender-matched with healthy classmates who served as controls. In the current study, we utilized a machine learning approach based on the elastic net to create a stable sparse classifier. Interestingly, the classifier was driven predominantly by nutrition group differences in alpha activity in the lingual gyrus. This structure is part of the pathway associated with generating alpha rhythms that increase with normal maturation. Our findings indicate that the PEM group showed a significant decrease in alpha activity, suggestive of a delay in brain development. Childhood malnutrition is still a serious worldwide public health problem and its consequences are particularly severe when present during early life. Deficits during this critical period are permanent and predict impaired cognitive and behavioral functioning later in life. Our EEG source classifier may provide a functionally interpretable diagnostic technology to study the effects of early childhood malnutrition on the brain, and may have far-reaching applicability in low resource settings.

## Introduction

Childhood malnutrition continues to be a serious health problem worldwide and is the primary cause of morbidity and mortality in children under 5 years of age (UNICEF et al., 2017). Protein Energy Malnutrition (PEM) in particular is prevalent among infants aged 6 months to 5 years old and critically impacts brain and cognitive development (Morgane et al., 1993; Galler et al., 1996; Black et al., 2017). The Barbados Nutrition Study (BNS) is a unique 45+ year longitudinal cohort study that has followed individuals with histories of moderate-severe PEM limited to the first year of life and healthy controls who were classmates of the PEM participants. This study has documented cognitive and behavioral problems (Galler et al., 1983a, b, 2010) including poor attention, impaired school performance and increased conduct disorder as well as depressive symptoms over the lifespan. Recent evidence suggests that early childhood malnutrition leads to epigenetic changes that impact the next generation (Peter et al., 2016).

We recently recovered EEG data that was collected in BNS participants in 1977–1978 at ages 5–11 years. EEG analyses using qEEGt (Taboada-Crispi et al., 2018) showed the following results in study participants with histories of childhood malnutrition: (1) increased theta activity (3.91–5.86 Hz) in electrodes T4, O2, Pz and in the supplementary motor area (SMA); (2) decreased alpha1 (8.59–8.98 Hz) in fronto-central electrodes and sources of widespread bilateral prefrontal area; (3) increased alpha2 (11.33–12.50 Hz) in temporo-parietal electrodes as well as in sources in central-parietal areas of the right hemisphere; and (4) increased beta (13.67–18.36 Hz), in T4, T5, and P4 electrodes and decreased in bilateral occipital-temporal regions of PEM versus control groups. Earlier EEG studies in children with histories of childhood malnutrition (e.g., Bartel et al., 1979) similarly found increased slow wave rhythms (theta band), as well as decreased alpha. The effect of early malnutrition on brain maturation and delayed brain development has also been reported in animal models of early malnutrition (Bronzino et al., 1999).

Studies directly examining brain function in individuals with histories of childhood malnutrition are limited. Although neuroimaging techniques such as MRI have been used to identify a neural signature of early PEM (Ivanovic et al., 2002), these techniques are not feasible for the development of scalable screening programs in low resource settings where PEM is most prevalent. They are also costly and have the disadvantage of low throughput.

Consequently, attention has shifted to EEG studies for identifying brain signatures of malnutrition. A prime candidate for this purpose is tomographic quantitative electroencephalography (qEEGT), which quantifies the EEG rhythms via its frequency spectrum—the power in the signal at each frequency bin and each channel. However, standard EEG studies have limitations, especially because of their reliance on scalp recording which is problematic for pinpointing anatomical substrates, physio-pathological explanations and the relationship to the animal literature. It is therefore preferable to carry out EEG Source Imaging (ESI) (He et al., 2019) for increased biological validity. Our earlier report (Taboada-Crispi et al., 2018) partially addressed these issues by:

1. Employing z spectra: each log spectral value has the age appropriate mean and divided by the age appropriate standard deviation (as encoded in regression equations obtained from the first wave of the Cuban Human Brain Mapping Project) (Szava et al., 1994). This ensured correction for normal brain age related variance.
2. Utilizing our novel machine learning technique (Bosch-Bayard et al., 2018) to identify a stable neural signature with high classification accuracy of EEGs to distinguish children with PEM versus controls.
3. Interpreting the neural signature by identifying their anatomical substrate differences with our ESI method Variable Resolution Electrical Tomography (VARETA) (Bosch-Bayard et al., 2001).

However, Taboada-Crispi et al. (2018) were not able to develop a classification procedure for malnutrition based on ESI measures. This was due to the extremely large number of highly correlated variables produced by VARETA. This method is based on a Bayesian inference structure which does not have a built-in variable selection procedure. To overcome this type of limitation we developed a new ESI method that guarantees sparse sets of active sources: Brain Connectivity Variable Resolution Electromagnetic Tomographic Analysis (BC-VARETA) (Paz-Linares et al., 2017, 2018, 2019; Gonzalez-Moreira et al., 2018). By leveraging the graphical lasso procedure (Friedman et al., 2010), we simultaneously estimate source activity and connectivity, thus producing a much sparser and decorrelated set of measures. This research paves the way for classification procedures based on EEG source spectra. For a recent review on ESI and connectivity see He et al. (2019).

In the current paper, we report the results of using BC-VARETA ESI to document the effects of early childhood PEM on brain function. We used BNS archival EEG data collected at ages 5–11 years (Ahn et al., 1980; Galler et al., 1983a, b) to identify a stable machine learning classification scheme to distinguish children with PEM versus controls based on EEG source spectra.

## Materials and Methods

### Barbados Nutrition Study Sample

The two nutrition groups were selected as follows:

1. PEM: Children born between 1967 and 1972 in Barbados and diagnosed with protein-energy malnutrition (PEM) in the first year of life (*n* = 129, 52 females, 77 males);
2. Controls: Healthy classmates (*n* = 129, 52 females, 77 males), matched by age ± 3 months, gender and handedness.

Inclusion criteria for PEM and control children were, as follows: (1) birth weight > 2500 g; (2) Apgar score > 8 at birth; (3) no birth complications; and (4) no encephalopathic events in childhood. The PEM group experienced a single episode of Grade II or III PEM (Gomez et al., 2000) in the first year of life based on clinical diagnosis at the time of admission to the Queen Elizabeth Hospital. The control group met the same inclusion criteria as the PEM group but did not have a history of PEM. Final selection was based on parental consent and access to birth and preschool health records. All PEM children were enrolled in a national program (NIP- Nutrition Intervention Program)- that provided subsidized food, maternal nutrition education, regular home visits, a pre-school nursery, and health care from the time of hospital discharge until 12 years of age (Ramsey, 1979), ensuring that no child had further episodes of malnutrition.

Written informed consent was obtained from all participants. Approval for this study was granted by the Ethics Committee of the Ministry of Health, Barbados, the Judge Baker Children’s Centre Human Research Review Committee (Assurance No. FWA 00001811) and the Massachusetts General Hospital IRB (2015P000329/MGH). Participants were compensated for their time and travel to and from the BNS research center.

### EEG Data Acquisition and Preprocessing

A complete description of the EEG procedures has been previously reported in Taboada-Crispi et al. (2018). Briefly, EEGs were recorded in 1977–1978 when the BNS children were 5–11 years of age by trained staff at the Barbados Nutrition Centre, who were blinded to the child’s nutritional history. A designated room was available for EEG recording. All participants were instructed to sit in a comfortable half recliner chair and to close their eyes but not to fall asleep. A custom-designed digital electrophysiological data acquisition and analysis system (DEDAAS) was constructed by Prof. E. Roy John at the Brain Research Labs, NYU (Thatcher and John, 1977) and was used to acquire the EEG data. The DEDAAS front-end consisted of 24 solid-state EEG amplifiers. The output of the amplifiers was fed through a 12-bit A/D converter with a sampling frequency (*f*_s_) of 100 Hz into a PDP-11 minicomputer that calibrated the amplifiers and checked the electrode impedances automatically. Simultaneous monopolar recordings were obtained of the 10/20 International Electrode System (Fp1, Fp2, F3, F4, C3, C4, P3, P4, O1, O2, F7, F8, T3, T4, T5, T6, FZ, CZ, and PZ) system, all referenced to linked earlobes. Data was stored on digital tape at the Brain Research Lab, New York University until 2016 when it was shared with our group (courtesy of Prof. Leslie Prichep). A total of 258 digital resting state EEG recordings were collected but only 137 raw EEG files were recovered in 2016 for the analysis. The original raw dataset was converted to EEGLAB (Delorme and Makeig, 2004) and PLG^1^ format for further processing.

Two neurophysiologists carried out quality control using visual inspection via time and frequency domain tools. Artifacts derived from oculo-motor and facial movements were eliminated use the AAR plug-in from the EEGLAB 13.6.5b toolbox described by De Clercq et al. (2006) and Gomez-Herrero et al. (2006). In sum, 29 recordings displayed somnolence and were excluded from this study, leaving a final dataset of 108 recordings (of the original 258).

### Source Space Analysis Using BC_VARETA

#### Source Imaging Technique

For the usable data, 1 min of artifact free EEG was obtained for all channels and was divided into quasi-stationary segments that were 2.56 s long. This yielded a total of *k* = 1,⋯,*T* = 24 windows. Each window was subjected to a Fourier transform each frequency ω, and segment *k*_and yielded_ a vector of complex Fourier coefficients V_k_(ω). In the current report, we attempt to estimate the sources of these vectors.

As noted in the introduction, we used Variable Resolution Electrical Tomography (VARETA) as the Electrophysiological Source Imaging (ESI) technique in our previous study on malnutrition (Taboada-Crispi et al., 2018). VARETA has been extensively used in clinical studies (Bosch-Bayard et al., 2001) but has two main disadvantages for using its features as variables for classifiers: lack of sparseness and also “activation leakage”– the spillover of estimated activity from the actual active cortical voxels to other sites due to unavoidable ESI reconstruction errors.

We overcame these problems in the current report using another ESI, the Brain Connectivity VARETA or BC-VARETA (in place of VARETA), which consisted of two stages:

1. “Screening” of the entire voxel space to retain only those voxels with possible activation. This is carried out with our new method for activation detection via model evidence maximization of the non-linear-univariate ENET-SSBL model (Paz-Linares et al., 2017). In the cited paper we reported that this new technique produced activation maps with the highest sparsity and least leakage of many state-of-the-art ESI methods.
2. The second stage consisted of an improved estimation of activation, achieved with simultaneous estimation of source connectivity (Paz-Linares et al., 2018, 2019). This approach substantially improves the connectivity estimation as well as source activity due to their mutual interdependence.

We now formally summarize this second stage for ease of reference. Scalar quantities are denoted by lower case capital letters (*x*), vectors by lowercase bold ones (*x*), and matrices are indicated by bold upper-case notation (*X*). Observed quantities will be denoted by Latin script and latent variables by Greek script. The usual conventions are followed: e.g., *x*^*T*^,*X*^−1^ are, respectively the transpose of a vector and the inverse of a matrix. Since we are working in the frequency domain we assume that all vectors *x* are considered as complex (*x* ∈ *ℂ*^*p*^), and distributed as independent random vectors with a Circularly Symmetric Complex Multivariate Normal probability density function, that is $x∼N_{q}^{\mathbb{C}}\,\left(\mu ,\Sigma \right)$ with dimension *q*, mean μ, and covariance matrix Σ

The frequency domain resting state EEG is modeled as:

(1) $$v_{k}\,\left(\omega \right)=L\,\iota_{k}\,\left(\omega \right)+e_{k}\,\left(\omega \right)$$

where *v*_*k*_(ω) ∈ *ℂ*^*m*^ is the complex EEG Fourier coefficient vector of dimension *m*, frequency ω and the *k*^*th*^ segment. Also *e*_*k*_(ω) is the corresponding sensor noise while *L* is the lead field matrix defined on the cortical surface for q. Finally, the sources of the EEG are denoted by the vector ι_*k*_(ω) ∈ *ℂ*^*q*^, where q is the number of sources. Since processing of each frequency ω is independent this argument will be dropped henceforth. The model is additionally specified by the following hierarchical Bayesian model for each subject:

(2) $$\begin{array}{c} a)v{}_{k}∼N_{q}^{\mathbb{C}}(L\iota_{k},\sigma_{e}^{2}R)\\ b)e{}_{k}∼N_{q}^{\mathbb{C}}(0,\sigma_{e}^{2}R)\\ c)\iota {}_{k}∼N_{q}^{\mathbb{C}}(0,\Theta_{\iota \,\iota }^{-1})\\ d)\Theta {}_{\iota \,\iota }∼e{}^{-\lambda \,{||\Theta_{\iota \,\iota }||}_{1}} \end{array}$$

In Equation [2 (a) and b] codify the observation equation (1) with the sensor error variance $\sigma_{e}^{2}\,R$ and *R* known. Line c) specifies a prior distribution for the sources with a covariance matrix $\Theta_{\iota \,\iota }^{-1}=\Sigma_{\iota \,\iota }$ with the inverse covariance (or Precision matrixΘ_ιι_ and cross-spectral matrix ∑_ιι_. Θ_ιι_is also known as the matrix of partial covariances and is usually assumed independent in most current. The novel feature of BC-VARETA is that it estimates Θ_ιι_in a data-driven fashion by assuming it is, in turn, a sample from a Gibbs distribution with general penalty function *P*(Θ_ιι_), here an L1 norm. All parameters are estimated via Expectation Maximization optimization of the model evidence. In summary BC-VARETA finds estimators of the Estimators of the cross-spectra ${\hat{\Sigma }}_{\iota \,\iota }\,\left(\omega \right)$ for each frequency. The diagonals of these matrices are the power spectra in the sources. Further technical details are in the cited papers and the software is available at https://github.com/CCC-members/BC- VARETA_Toolbox.

#### Specific Source Analysis

BC-VARETA was used to analyze the artifact-free EEG dataset for each participant to obtain the log source spectra at each of 6003 cortical sites and all 48 frequency components within a range of 0.1–19 Hz. These were then summarized by averaging over the areas of the AAL atlas of the MNI (Mazziotta et al., 2001). An approximate Lead Field matrix used for the source-space analysis for all subjects was obtained with Brainstorm software^2^ for 19 sensors defined on the 10–20 system and co-registered using the MNI Average Brain template subject anatomy. This approach of using an average lead field (across the sample) was experimentally tested (Valdés-Hernández et al., 2009) resulting in the most convenient tool in research studies demanding EEG source localization when MRI are unavailable. The atlas employed by us to identify the neural structures is available as a toolbox for SPM at http://www.gin.cnrs.fr/AAL2. To further summarize the number of frequencies and reduce the final number of variables, we grouped the bins of frequencies using the broad band parameter, according to the IFCN Guidelines (Nuwer, 1997) and proposed the following frequency bands: delta (1.5–3.9 Hz), low theta (4–5.4 Hz), high theta (5.8–7.4 Hz), low alpha (7.5–9.4 Hz), high alpha (9.5–12.5 Hz), low beta (12.8–14.9 Hz), and high beta (15–19.14 Hz).

### Stable Sparse Biomarkers Detection (SSB)

The EEG spectral signatures obtained from BC-VARETA are used here to identify biomarkers that discriminate between the two nutrition groups using the EEG activity. In what follows we denote the spectral estimators for each frequency band/location as *s*_*i*,*j*_*i* = 1,⋯*N*;*j* = 1,⋯*p*, where *N* is the total number of subjects and *p* is the total number of features (potential biomarkers) to be explored. For this purpose, we use the SSB methodology (Bosch-Bayard et al., 2018; Chiarenza et al., 2018) which is a classification procedure that extract a minimal set of features, in a high dimensional problem, by providing a classification equation with a high predictive power and stability. SSB specially deals with the case where the number of variables is high (*p* = 294,147 in our case) and the number of observations is relatively small (*n* = 108). In such *p* > > N situations it is possible, by chance, to achieve classification equations with spuriously high accuracy. Nevertheless, slight changes in the training set can lead to quite different feature selection and classification rates.

To protect against this problem, the sparse stable biomarker (SSB) proceeds in two steps.

#### First Step: Selection of a Stable Set of Predictors

This is done by a resampling methodology that repeatedly and randomly splits the data (in our case 500 times) with 70% of the data in a training set and 30% in a test set. With the generation of each random pair of training and testing sets the following operations are carried out:

(A) The indfeat procedure (Weiss and Indurkhya, 1998) winnows out promising classification variables in the training set.

(B) An even smaller set of predictors is selected from the training set by means of the elastic net regression (GLMNet) (Hastie et al., 2016) to select a classification equation (Zou and Hastie, 2005; Friedman et al., 2010). The model is described by the equation (3):

(3) $$\min_{\varphi_{0}\in ℜ,\varphi \in ℜ}\left[\frac{1}{2\,N}\,\sum_{i=1}^{N}{\left(y_{i}-\varphi_{0}-x_{i}^{T}\,\varphi \right)}^{2}+\lambda \,P_{\gamma }\,\left(\varphi \right)\right]$$

Here *N* is the number of subjects, *x*_*i*_ ∈ ℜ.*x*_*i*,*j*_ = *log*⁡(*s*_*i*,*j*_)*x*_*i*_ ∈ *ℜ* observations of subject *i*, and *y*_*i*_ ∈ *ℜ* is the label group of subject *i*; *φ*_0_ ∈ ℜ,*φ* ∈ ℜ are the model parameters; γ is the regularization parameter; *p* is the number of variables in the model; and

(4) $$P_{\gamma }\,\left(\varphi \right)=\left(1-\gamma \right)\,\frac{1}{2}\,{||\varphi ||}_{2}^{2}+\gamma \,{||\varphi ||}_{1}$$

The penalty *P*_γ_ in equation (4) is known as the elastic-net norm (Zou and Hastie, 2005). To understand its behavior, note that the ${||\varphi ||}_{2}^{2}$ norm induces regressions that behaves well for high dimensional regressions but that tend to spread out coefficient weights among highly correlated variables. On the contrary, the ||φ||_1_ norm produces the “lasso regression” which is indifferent to highly correlated predictors and tries to select only one thus inducing sparsity. The elastic-net reaches a compromise between the ridge and the lasso, the relative contributions being determined by the γ and λ parameters. Since these parameters are selected by cross-validation (based on the test set), in any specific case, the sparsity of the solution will be data-driven. The set of predictor variables selected at this repetition is recorded.

Finally, after all repetitions, a stable set of predictor variables is obtained by retaining only those variables that are selected in at least 50% of repetitions.

#### Second Step: Evaluation of the Stable Predictor Set

In a totally independent set of resampling experiments, the sensitivity and specificity of the classification equation is evaluated. In an earlier report (Bosch-Bayard et al., 2018), a ROC methodology was introduced which also guarantees stability and robustness. Again, the total sample is repeatedly and randomly split into two samples 70% of the data for the training set and 30% for the out of sample test set. For each repetition the following operations are carried out:

(A) A GLMENT classifier is obtained from the training set as using a similar procedure as in the first step (B) above.
(B) The area (AUC) and partial areas under the ROC curve (pAUC) is calculated on the training set and stored.

The AUC and pAUC values are used to generate kernel empirical distribution functions for these measures. We use the median of these distributions as an estimate of the true underlying measures accuracies of the classification procedure. In the case of the pAUC, these are evaluated for the False Positive Ratios 0.1 and 0.2 and transformed to the standardized partial Area under the ROC curve (spAUC) as described by McClish (1989) to facilitate comparisons. Note that SSB described in this section is not to be confused with the ENET-SSBL procedure (Paz-Linares et al., 2018).

### Age Adjusted Classifier and Interpretation

Note that in our previous report in the BNS participants (Taboada-Crispi et al., 2018), gender and age were included as covariates in the EEG analyses. However, only age was found to be significantly correlated with the EEG and is therefore the primary covariate included in the analysis described below. In our previous work with VARETA we pre-process the log source spectra by the z-transformation: to partial out the variability due to normal age changes. Normative data is not yet available for BC-VARETA. Instead, we obtained an age adjusted classifier by introducing both age and the interaction (product) of age and log source activity as potential biomarkers.

(5) $$\min_{\varphi_{0}\in ℜ,\varphi \in ℜ}\left[\frac{1}{2\,N}\,\sum_{i=1}^{N}{\left(y_{i}-\varphi_{0}-x_{i}^{T}\,\varphi -a\,g\,e_{i}\,x_{i}^{T}\,\psi \right)}^{2}+\lambda \,P_{\gamma }\,\left(\varphi ,\psi \right)\right]$$

Where the notation is the same as in equation (3) with the additional parameter vector ψ which represents the slope of the age-dependent classifier, with *age*_*i*_ the age of subject *i*.

Since the SSB procedure provides the biomarkers and their coefficients in the classification equation, we performed an additional t-test analysis between the two groups, using the contrast malnutrition (PEM) versus Control group in order to determine the direction of the group differences. In this case, the negative sign indicated lower activation of PEM and the positive indicated higher activation of the PEM group. This last analysis in included for illustrative purposes only and is not part of the classification procedure.

## Results

### Demographic Characteristics of the Sample

Table 1 summarizes the demographic characteristics of the study participants. There were no nutrition group differences in gender, age or handedness. The table shows significant differences between the PEM and control groups in IQ, academic performance and ecology at 5–11 years, as previously reported in the full sample (Galler et al., 1983a, b). This subsample retains the age/sex balance of the original 1977-1978 cohort.

**TABLE 1:** Demographic characteristics of the sample.

	**PEM**	**Control**	***t*-test/χ2**	***p*-value**
*N*	46	62		
Males [*N* (%)]	28 (60.9)	34 (54.8)	0.39	0.531
Age (years)				
- Males	8.5 ± 1.9	8.6 ± 1.7	0.14	0.888
- Females	7.9 ± 2.0	8.5 ± 1.9	1.01	0.318
Handedness [N left (%)]	4 (8.7)	3 (4.8)	0.65	0.456
Childhood Ecology	−1.14 ± 0.89	−0.14 ± 0.81	6.01	< 0.0001
WISC Full-Scale IQ	88.9 ± 12.9	105.2 ± 11.9	6.62	< 0.0001
School Performance (1–5)	3.1 ± 1.06	4.3 ± 0.88	6.20	< 0.0001

*Data are presented in mean ± SD or %; Age (in years at the moment of the EEG recordings). The differences between groups were tested using Chi-square and *t*-test.*

### Age Adjusted Classification of PEM vs. Control Children Using BC-VARETA Sources

A linear mixed-effects model testing influence of age and sex on the source variables (Chung et al., 2010) showed no effect of sex but did show a significant effect of age. To deal with this an age-adjusted classifier was developed and is described below. The regions and frequency bands selected as stable classifiers are listed in Table 2. The third and fourth column show the % of times selected as a classifier during the randomization procedure for the age-independent coefficients. The fifth and sixth column show the same information for the age-dependent coefficients ψ.

**TABLE 2:** Regions and frequency bands selected as stable classifiers.

**Region of AAL atlas**	**EEG Frequency Band**	**% times selected as classifier**		** Regression Coefficients**	
		**ϕ**	**ψ**	**ϕ**	**ψ**
Middle Temporal Gyrus Left (MT.L)	High Theta θ_*H*_	78.72	75	–0.28	–0.02
Inferior Frontal Gyrus Orbital Right (IFO.R)	Low Alpha α_*L*_		54.55		–0.006
Lingual Gyrus Right (LING.R)	Low Alpha α_*L*_	50.6		228.26	
Cuneus Right (CUN.R)	High Alpha α_*H*_	75	81.71	0.18	0.03
Pre-Central Gyrus Right (PRECG.R)	High Alpha α_*H*_	60.82	51.22	–1.18	–0.06
Lingual Gyrus Right (LING.R)	High Alpha α_*H*_	52.44	53.66	198.02	31.29
Superior Temporal Gyrus Left (ST.L)	Low Beta β_*L*_	50.59		–0.20	
Middle Occipital Gyrus Right (MO.R)	Low Beta β_*L*_	75.42	53.33	–0.094	–0.006
Superior Medial Gyrus Left (SMG.L)	Low Beta β_*L*_	57.83	53.57	–0.19	–0.013
Inferior Temporal Gyrus Left (ITG.L)	High Beta β_*H*_	76.4	56.25	–0.19	0.009

*From left to right: anatomical region, frequency band% times selected as a classifier, and the actual regression coefficients of the elastic net classifier. ϕ Are the coefficients for each variable for those independent of age, and ψfor those that reflect the interaction with age.*

**FIGURE 1:**
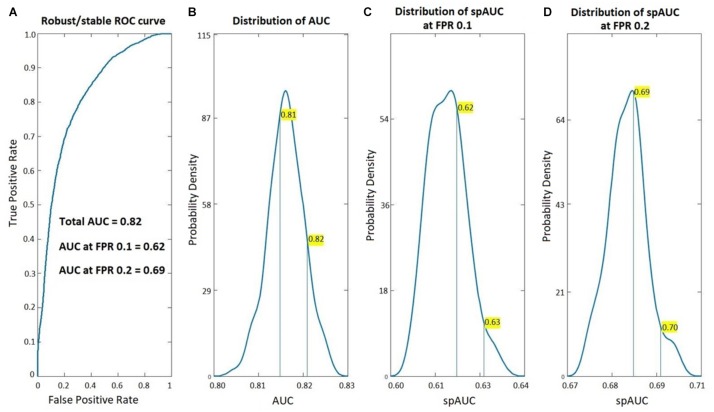
Classification accuracy of the age-adjusted PEM vs. Control Classifier. ROC analysis of the age-adjusted classification between Children with PEM and Controls based on the BC-VARETA. Electrophysiological Source Imaging techniques. On the left, **(A)** the full ROC curve, on the right **(B–D)** the probability density functions (estimated from the resampling cross validation) of the area under the ROC curve (AUC) for the full curve **(B–D)** the standardized partial AUC (spAUC) at 0.1, and 0.2 false positive ratio cut-off points respectively.

Figure 1 shows the ROC analysis of the age-adjusted classification procedure based on these coefficients BC VARETA to distinguish between the EEGs of both groups (PEM vs. Control). Note that this is the same EEG dataset reported (Taboada-Crispi et al., 2018). As can be seen the classification accuracy is quite high with the using EEG sources calculated with the VARETA procedure. As can be seen the classification accuracy is quite high with the area under the curve 0.82. The figure also shows that the estimated probability density for the AUC values from the randomized subsamples is quite far away from 0.5 (chance classification). This is also true for the standardized partial area under the ROC curve (spAUC) at both the 0.1 and 0.2 false positive rate cut-off points.

**FIGURE 2:**
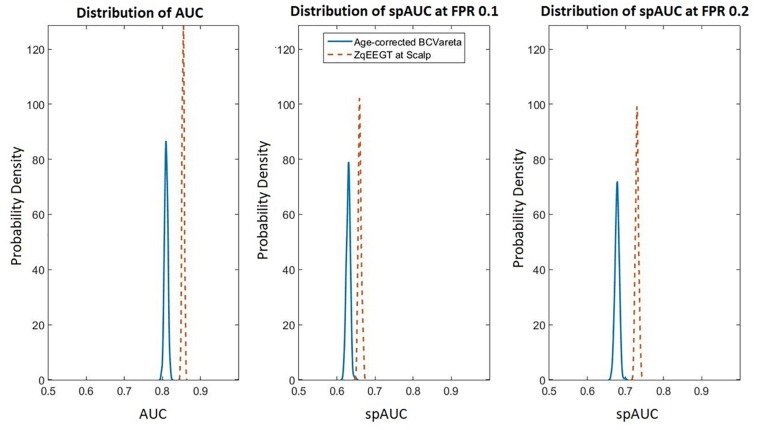
Comparison of scalp based and source-based BC-VARETA classification accuracy. As in Figure 1, the probability density functions of the AUC are shown for the full curve **(left)**, the spAUC at 0.1 **(center)**, and at 0.2 **(right)** false positive probability cut-off points. The blue (solid) lines correspond to the age-adjusted BC-VARETA classifier, while the red (dashed) lines correspond to the scalp qEEG based classifier. The scalp-based classification performed slightly better than the classifiers at the sources.

Figure 2 compares the resampling-based probability densities for the AUC and spAUC as in Figure 1 for the age adjusted classification based on BC-VARETA sources and superimposed, for purposes of comparison, the same curves for the classifier based on scalp qEEG measures previously presented in Taboada-Crispi et al. (2018). Both are very high, with a slight advantage for the scalp-based qEEG measures.

**FIGURE 3:**
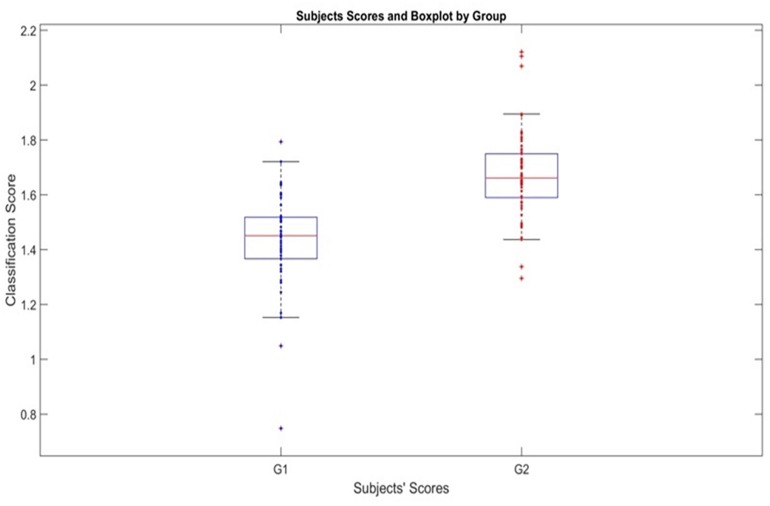
Classification scores produced by the age-adjusted classifier for PEM and Control Groups. Boxplot showing the scores *t*_*i*_ of subjects for both groups using the individual classification scores. G1 is Malnutrition group (PEM) and G2 Control group.

The actual effectiveness of the classification is shown in Figure 3 with the boxplot of the individual classification scores

(6) $$t_{i}=\varphi_{0}+x_{i}^{T}\,\varphi +a\,g\,e_{i}\,x_{i}^{T}\,\psi =\sum_{j=1}^{p}x_{i,j}\,d_{j}\,\left(a\,g\,e_{i}\right)$$

Which is based on the age adjusted elastic net classifier (5). Note that *d*_*j*_ (*age*_*i*_) is the age-adjusted regression coefficient.

There is a clear separation provided by the stable age-adjusted classifier. Note that this classification is based on the test set, not the training set for the median value of the AUC curve density.

**FIGURE 4:**
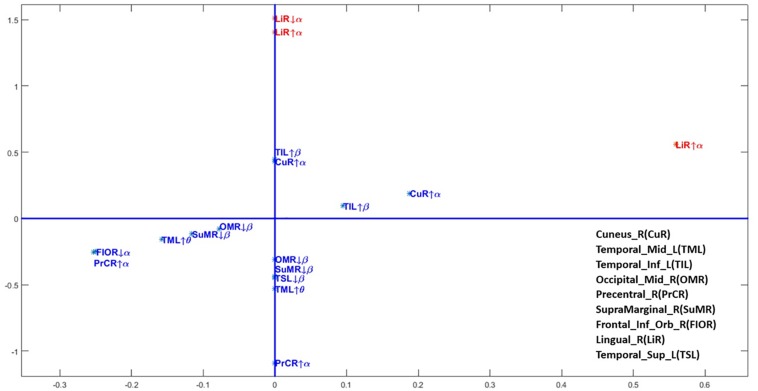
Scatterplot of the coefficients of the age-adjusted classification regression equation. The regression coefficients for each frequency band and source anatomical region included in the age-adjusted classifier. Axis transformed by a sqrt root function to improve visualization. On the horizontal axis the coefficient ϕ_*j*_ for the interaction with age and on the vertical axis the age intendent coefficients ψ_*j*_. This is the same information as in Table 2. Note that due to the disparity of scales the alpha activities in the lingual gyrus were also further divided by a factor of 100.

Figure 4 shows the values of the coefficients (also in Table 2). In this figure positive for both coefficients indicate that increased activity (taking into consideration age or not) drives the score toward the control group. Due to the disparity in the values of the coefficients a square root transformation and rescaling of the alpha activity in the right lingual gyrus were applied. Thus, classifier is dominantly driven by low and high alpha activity in the right lingual gyrus, which is part of the occipital lobe.

**FIGURE 5:**
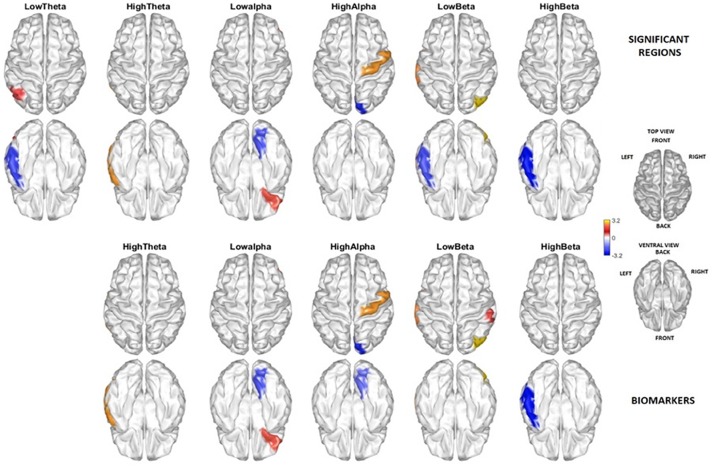
Brain areas and frequencies contributing to the discrimination between PEM and Control. The two rows above show the regions by frequency bands with significant *t*-test group differences (threshold corrected by permutations) contrasting PEM vs. Control. Highlighted are those areas with a *t*-test in a range of exceeding (red) and below (blue) the permutation selected threshold for *p* < 0.05. The two lower rows show the brain regions and frequencies selected as biomarkers. There is a quite good correspondence between the two independent results, except that no biomarker was selected in the Delta band by the classification procedure.

Figure 5 (two upper rows) displays t-test comparisons (threshold selected by permutations) between the source activity of PEM vs. control groups. Areas colored in blue indicate negative values, for those frequency bands and structures in which PEM has significantly less activation than control. Red indicates significant positive t statistics, where PEM has excess activation when compared with controls. This figure is only shown to allow comparison with similar results using VARETA (Taboada-Crispi et al., 2018).

The areas selected by the age-adjusted classifier are shown in the lower two rows of the figure. Note that all the areas included in the age-adjusted classifier are significant other than the lingual gyrus hi-alpha which is just below the permutation threshold.

## Discussion

### Classification Accuracy

In this paper we report a new classification procedure that uses source activity estimated using the BC-VARETA procedure, to delineate neural effects of exposure to protein energy malnutrition (PEM) in the first year of life. As seen in Figure 1, the area under the ROC curve is well above chance level. Importantly, it is necessary to limit the False Positive Rate (FPR) to either 0.1 or 0.2 to obtain consistent and high classification rates. Such low FPRs are of practical importance, since in screening programs an excess of false positives might overload health systems. The good performance of our classifiers at low FPR protects against this.

### An Alternative View of Frequency Bands and Source Locations

The regression coefficients in Table 2 are interpreted, not in terms of the log transformed power at the sources but in terms of the power. If one takes the exponent of the classification equation (6) then the classification score is the product of terms:

(7) $$\exp\left(t_{i}\right)=\prod_{j=1:p}s_{i,j}^{d_{j}\,\left(a\,g\,e_{i}\right)}$$

with *t*_*i*_ the classification score for subject *i*, *s*_*i,j*_ the spectrum for *i* in frequency band/anatomical location *j*. Note that this (7) is a product of the source activations with the age-dependent regression coefficients *d*_*j*_(*age*_*i*_) as exponents. This formula provides a data-driven generalization of previous power ratios that were previously popular. For example, the α/ϑ ratio for any given sources is obtained by setting the *d*_α_(*age*_*i*_) = 1, and *d*_θ_(*age*_*i*_) = −1, irrespective of the data.

The resulting classifier is mainly driven by alpha activity in the right lingual gyrus. The signs of the age dependent regression coefficients indicate that low and high alpha activity in this area contribute to classification of nutrition status in the first year of life. Specifically, low alpha values in the lingual gyrus are an indicator of a history of PEM. This is in agreement with the significant differences between the two nutrition groups, as confirmed by *t*-tests (Figure 5).

### Comparison With the Previous Classifier Using Scalp qEEG Variables

We previously used the Sparse Stable Biomarker (SSB) selection method to obtain a classifier to differentiate between PEM/Control for this same data set (Taboada-Crispi et al., 2018). In our earlier paper, the potential biomarkers were the log EEG spectra at the scalp (topographical level) which yielded an AUC of 0.83, nearly equivalent to our current result. Due to the use of a randomization sampling scheme, the estimates vary somewhat, ranging from 0.81 to 0.86 for the qEEG classifier.

As mentioned previously, it was impossible to use a source-based classifier in Taboada-Crispi et al. (2018) due to the high dimensionality and correlation of VARETA sources, a limitation which we have overcome in the present paper. The new classifier achieves similar accuracy compared to that reported previously, allowing us to now identify the areas that most differentiate PEM from controls with high confidence. However, our classifier selects the minimal subset needed to accurately identify children with early malnutrition and this is only a small portion of the widespread areas affected by PEM. As such, a more complete analysis follows.

### Physiological Interpretation of PEM-Control Differences

The neurophysiological impairments associated with early PEM can be permanent, often accompanied by widespread neurological disturbances involving sensory-motor activity, learning, memory, consciousness, cognition and emotion (Guedes, 2011). The BNS study is unique because the participants experienced a single episode of malnutrition limited to the first year of life. In order to identify all areas affected by malnutrition, *t*-tests at the source level (threshold corrected by permutations) compared participants with PEM with Controls (Figure 5). Many frequency bands/areas identified in this study were previously reported (Taboada-Crispi et al., 2018).

Notably, there are widespread changes in alpha activity; alpha is decreased in previously malnourished participants in the right inferior fronto-orbital area, and increased in the right precentral gyrus. However, a novel finding is the marked decrease of alpha activity in the right lingual gyrus for the PEM group made possible only by the improved localization capability of BC-VARETA. This is the single feature which predominantly accounts for individual classification of participants. These findings lend further support to our hypothesis (Taboada-Crispi et al., 2018) that early PEM impacts timely cortical myelination thereby causing a delay in the development of the alpha rhythm. This conclusion is supported by several lines of evidence:

1. Cortical rhythmic activity depends critically on thalamo-cortical pathways and the inhibitory feedback of the thalamic reticular nucleus (Llinás and Steriade, 2006). This has been modeled in detail previously (Valdes et al., 1999; Valdes-Sosa et al., 2009). These models predict an increase in the peak alpha frequency dependent on the set of parameters defining the thalamo-cortical loop. The specific role of axonal delays is also considered in Douglas and Douglas (2019).
2. A previous study of 300 normal subjects from the Cuban Human Brain Mapping Project database showed that the peak alpha frequency in normal subjects depends on the microstructure of thalamo-cortical pathways in the optic radiation, thus supporting our neural mass model explanation (Valdés-Hernández et al., 2010).
3. Extensive normative studies by our group have shown that the peak alpha frequency increases from the high theta band to the typical alpha band over the lifespan (5–97 years). This is valid both for scalp EEG (Alvarez Amador et al., 1989) and sources (Bosch-Bayard et al., 2001). These changes in alpha are most pronounced in the first years of life.
4. Studies of myelin development resulting from longitudinal studies have demonstrated that normal brain development involves a linear increase in white matter from childhood to adulthood especially in the optic radiation (Almli et al., 2007; Laule et al., 2007; Dean et al., 2014). See especially Figure 5 FA changes for the OR in Dubois et al. (2014). For a review of related studies see Tau and Peterson (2010).
5. In the current study, PEM took place during the first year of life, the most significant period of myelination. This may have life-long lasting effects on alpha rhythm development, as evidenced by a slower peak alpha frequency.

### Limitations

**FIGURE 6:**
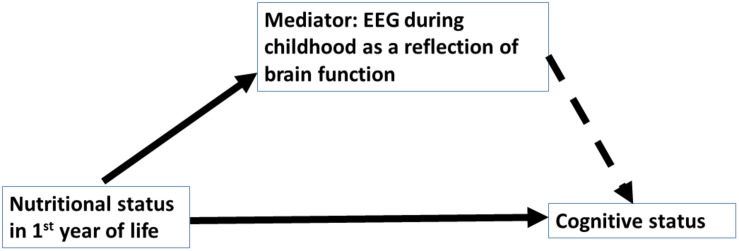
Conceptual model to study EEG sources as mediators. The current paper is part of a program to determine EEG biomarkers signaling neural mediators on the long-term effect of PEM in the first year of life on childhood cognitive performance. The solid arrows indicate confirmed paths: Nutrition- > cognitive variables (Galler et al., 1983a, b); Nutrition - > EEG (this paper). Dashed arrow indicates paths to be confirmed.

This study has several limitations:

1. The variables explored as classifiers are the source log Spectra estimated by BC-VARETA. An essential, but still difficult problem is the full incorporation of partial coherences (brain network information) in addition to source activations as classification variables. This will conceivably improve classification accuracy greatly. While the calculation of partial coherences is inherent to BC-VARETA, their use is challenging due to the need for Riemannian classification procedures mandated by the estimated quantities (Li et al., 2009).
2. Due to the novelty of the BC -VARETA technique and the statistical challenges involved with this procedure, a comprehensive calculation of multinational age dependent norms is being developed and will be tested in the future.
3. Importantly we are analyzing data in fixed frequency bands. Methods based on individualized moments of the spectra or decompositions into peaks (Pascual-Marqui et al., 1988; Valdés et al., 1992) could further serve to enhance the accuracy of the classification scheme since it would focus on peak alpha frequency. However, we have already shown elsewhere that the less computer intensive approach using fixed spectral bins can serve as a screening procedure for further analysis.
4. In Taboada-Crispi et al. (2018) we showed that visual inspection of the EEG by experts provided additional information about brain states in both PEM and control groups. These evaluations were based on “grapho-elements” (such as “sharp waves” whose shape exhibits non-linearly determined time/frequency phase relationships). A non-linear set of features obtained at the source level also needs to be explored in order to quantify this type of assessment (Valdes et al., 1999).
5. This study is only the first step in building a disease progression model in which EEG variables are explored as potential mediators of the long-term cognitive and behavioral outcomes of childhood malnutrition. Such a model could incorporate other environmental factors to identify individual trajectories of the evolution of brain states. Our immediate research agenda is shown in Figure 6 for detecting the mediation (through altered brain function) of the effect of early malnutrition on cognitive outcomes. With this paper we have identified the link: Nutritional status - > Functional alteration in brain structures. The analysis of the full model will be carried out in future studies.

## Conclusion

The effects of Protein Energy Malnutrition in the first year of life can be detected by an age-adjusted classifier based on the logarithms of the EEG source spectra. Basing the classifier on the source spectra allows for an anatomic interpretation of the classifier’s variables—in this case alpha activity in the lingual gyrus. Thus, we provide evidence that EEG sources can be an important component to consider as mediators in disease progression models that ultimately could provide lifelong predictions of cognitive development to optimize cost/effective health interventions.

## Data Availability Statement

The EEG datasets employed for this study can be obtained by request to the corresponding author (pedro.valdes@neuroinformatics-collaboratory.org) and the dataset regarding social, demographic, and psychological assessment to JG (Janina.galler@gmail.com) who is the co-founder and co-director of the Barbados Nutrition Study.

## Ethics Statement

The studies involving human participants were reviewed and approved by the Ethics Committee of the Ministry of Health, Barbados, the Judge Baker Children’s Centre Human Research Review Committee (Assurance No. FWA 00001811), and the Massachusetts General Hospital IRB (2015P000329/MGH). Written informed consent to participate in this study was provided by the participants’ legal guardian/next of kin.

## Author Contributions

PV conceived, organized and executed this project with the collaboration of MB and JG who is the co-founder and director of the 45+ year Barbados Nutrition Study. MB, JG, and PV were involved with the recovery of the EEG archival data. The initial EEG pre-processing was performed by AC and the sensors and EEG source analysis was performed by YG and QT under supervision of PR, FR, and DP. The statistical analysis was designed by PV together with JB-B and LG. The manuscript was prepared by MB, PV, and JG. AR contributed to the writing and final editing of this report.

## Conflict of Interest

The authors declare that the research was conducted in the absence of any commercial or financial relationships that could be construed as a potential conflict of interest.

## Footnotes

1 http://www.neuronicsa.com

2 https://neuroimage.usc.edu/brainstorm/
